# From Mechanistic Models to AI Synergy: A Roadmap for Predictive and Personalized Biology

**DOI:** 10.3390/biology15201859

**Published:** 2026-10-16

**Authors:** Letong Huang, Hongqin Sun, Yi Huang, Qingyi Gu, Hailong Zhang, Dingqin He, Wenyi Zhang, Siyu Chen, Delin Xu, Juanjuan Zhao

**Affiliations:** 1 Department of Medical Instrumental Analysis, Zunyi Medical University, Zunyi 563000, China; zmuhlt0530@163.com (L.H.); 15761977926@163.com (H.S.); wenyi.zhang666@outlook.com (W.Z.); 15298107773@163.com (S.C.); 2 Key Laboratory of Cancer Prevention and Treatment of Guizhou Province, Zunyi 563000, China; hailongzyolo@163.com; 3 Department of Clinical Medicine, Nantong University, Nantong 226019, China; 15208650346@163.com (Y.H.); 13382883117@163.com (Q.G.); 4 Special Key Laboratory of Oral Disease Research and High Education Institute in Guizhou Province, School of Stomatology, Zunyi Medical University, Zunyi 563000, China; 15722157976@163.com

**Keywords:** mathematical modeling, artificial intelligence, predictive biology, biological digital twins, explainable AI (XAI)

## Abstract

Two paradigms now drive the life sciences. One is the explanatory depth of mathematical modeling, the other the predictive power of artificial intelligence. For decades, mechanistic models have offered a rigorous, hypothesis-driven framework for dissecting biological processes, while AI has proven itself at finding patterns in the large, high-dimensional datasets of modern biology. Each has delivered major advances on its own. Together they promise a synthesis that could redefine the boundaries of biological inquiry. In this review we propose a three-part taxonomy of integration strategies, namely AI-accelerated mechanistic modeling, mechanism-informed AI, and hybrid models spanning one-directional sequential coupling and bidirectional iterative systems, and we survey their applications in systems and synthetic biology, pharmacology and drug discovery, epidemiology, and neuroscience. We then examine seven challenges that stand in the way: interpretability, data sparsity, causality, identifiability, computational scale, multimodal integration, and validation and generalization, and we close with a view of biological digital twins, causal discovery engines, and automated science. Our argument is that combining these approaches is the principal task for a new generation of interdisciplinary scientists who aim to build a predictive and mechanistic science of life.

## Simple Summary

Biology currently runs on two modeling traditions. Mechanistic models write down the processes a biologist believes are at work, so their variables and parameters carry meaning, but they are slow to build and hard to fit to the volume of data that modern experiments produce. Deep learning finds patterns in that data with far less human guidance, yet its predictions rarely explain themselves, and it depends on large labeled datasets. This review asks how the two can be combined rather than pitted against each other. We organize the literature into three strategies: AI that accelerates mechanistic modeling, mechanistic knowledge that constrains AI, and hybrid designs in which the two exchange information in a loop. Across systems and synthetic biology, drug discovery, epidemiology, and neuroscience, the strongest cases test whether the mechanistic component adds value, not merely whether the combined model scores well. We then examine seven obstacles—interpretability, data sparsity, causality, identifiability, computational scale, multimodal integration, and validation—and sketch a roadmap toward digital twins, causal discovery engines, and automated science. A shared vocabulary and clear evidence standards would help make biological models both predictive and comprehensible, which is what personalized medicine ultimately requires.

## 1. Introduction

Biological research is currently undergoing a historical transition from qualitative description to quantitative analysis [1]. The traditional paradigms of observation and classification are no longer sufficient to support the in-depth advancement of modern life sciences and are being replaced by a new research framework centered on high-dimensional data acquisition, mathematical modeling, and statistical inference [2]. This shift is particularly evident in contemporary medicine and pharmacology: drug development relies heavily on quantitative pharmacology to predict pharmacokinetic behaviors both in vitro and in vivo; pathological diagnosis pursues causal chain resolution based on multi-omics data; and the standardization of clinical treatment protocols depends on reproducible and quantifiable biomarker systems [3]. However, the inherent nonlinear coupling relationships and endogenous fluctuations within biological systems leave many phenotypic phenomena beyond the reach of linear reasoning or expert intuition, so that the integration of mathematical language and dynamical models into the theoretical framework of life sciences is needed to uncover the regulatory logic beneath observational data [4].

Against this backdrop, the large-scale intervention of artificial intelligence in biological research is not accidental: under the data-intensive paradigm of scientific discovery, it was inevitable [5]. At present, multi-omics data, from genomics and transcriptomics to proteomics and metabolomics, are growing exponentially, with a single experiment capable of generating millions to billions of feature variables [6]. Concurrently, the validation of biological questions often requires multi-level cross-comparisons across scales (molecular, cellular, tissue, and organismal), overloading traditional statistics and hand-crafted feature engineering [7]. Artificial intelligence, particularly architectures such as deep learning and graph neural networks, captures nonlinear interactions and latent dependencies among high-dimensional features well, enabling end-to-end extraction of effective representations from raw data. This gives efficient, scalable solutions to classic problems such as gene regulatory inference, drug repositioning, and phenotype-genotype association discovery [8].

Mechanistic modeling has made progress in many fields of biology in recent years, and its canonical forms are mechanistic by construction. Michaelis–Menten enzyme kinetics, Hodgkin–Huxley membrane models, compartmental epidemic models, and pharmacokinetic models each encode hypothesized biological processes as explicit variables and parameters. At the ecosystem level, mechanistic and statistical models have mapped the spatial distribution of Amazonian tree diversity [9] and quantified cumulative risks associated with environmental change [10]. Mathematical models, in depth and in breadth, have left a lasting mark on biological research.

The turn of the millennium changed biology. High-throughput technologies, from next-generation sequencing and mass spectrometry to high-content imaging, began generating data at an unprecedented scale [11]. The problem was no longer a lack of information but the opposite: too much data, too little knowledge drawn from it. That is where deep learning found its clearest application in biology [12].

Deep learning has since succeeded across many fields [13]. AlphaFold2 reached accuracy competitive with experiment on the CASP14 single-chain structure-prediction benchmark, a result widely regarded as a breakthrough on the long-standing protein-folding problem, reshaping structural biology [14]. In genomics, convolutional neural networks predict gene expression and chromatin features from DNA sequence alone [15]. In cell biology, AI-driven image analysis classifies phenotypes and traces lineages in microscopy data faster and more accurately than a human expert [16]. These models excel at learning complex patterns and making accurate predictions. Their downside is the “black box”: millions of unexplained parameters leave the biologist with a correct answer but no account of why, so the models generate few new, testable mechanistic hypotheses [17]. This opacity is usually blamed on the complex nonlinear connectivity of deep networks and on benign overfitting that resists interpretation. The practical obstacles are just as serious: large datasets, complicated loss functions, and the tension between generalization and interpretability make deep networks hard to understand and hard to train [18].

For years, two modeling cultures, one mechanistic and one data-driven, worked side by side, and which one a lab chose depended on the problem [19]. The most pressing questions in biology, however, need both [20]. Predicting how a patient will respond to cancer treatment requires the general mechanistic principles of tumor growth and, at the same time, the specific patterns hidden in that patient’s multi-omics data. Out of this realization an emerging paradigm has grown: the synergistic integration of mathematical modeling and artificial intelligence [21]. Recent single-cell technologies and AI-driven frameworks already exemplify it. They decipher cellular dynamics from time-series transcriptomic data [22], link single-cell resolution to clinical outcomes, and support multidimensional assessment of organoid models through automated image and omics analysis [23,24]. Data-driven AI and knowledge-driven mechanistic models can inform each other, and the strategies surveyed in this review build on that complementarity.

**Figure 1:**
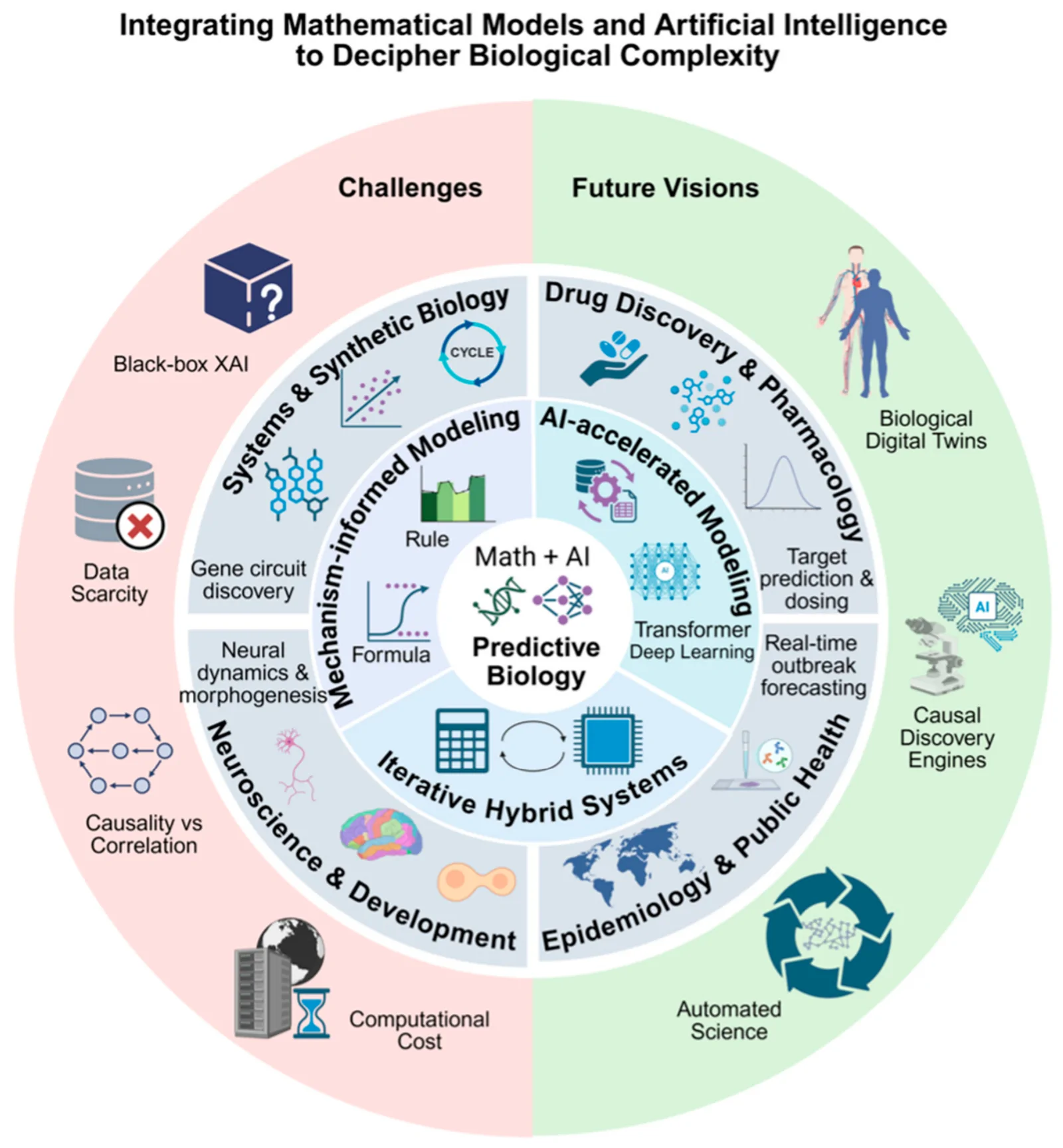
Conceptual roadmap of the review. The roadmap places the three integration paradigms along a continuum from purely mechanistic to purely data-driven modeling (left), lists representative enabling methods (center), and maps them onto four application domains (right). Definitions and detailed discussion are provided in Section 2 and Section 3.

In this paradigm, each approach compensates for the other’s weaknesses. Mechanistic knowledge supplies the foundation [25] and makes AI more data-efficient and more interpretable, while AI supplies the engine to explore the high-dimensional parameter and structural spaces that have long slowed mechanistic models down. The remainder of this review moves from foundational concepts to applications and, finally, to the challenges that remain (Figure 1).

Recent reviews have begun to close this gap, and we build directly on them rather than claiming an empty field. Puniya and colleagues map AI-driven mechanistic computational modeling and digital twins across biomedical applications [20], Noordijk and colleagues provide a systems-biology perspective on scientific machine learning [25], and broader reviews cover physics-informed machine learning [26] and simulation-based inference [18]. These works organize the literature mainly within a single domain or treat integration as an extension of one parent field. The present review makes three complementary contributions. First, we propose an operational three-part taxonomy: AI-accelerated mechanistic modeling, mechanism-informed AI, and hybrid models spanning sequential coupling and iterative closed loops, with explicit assignment criteria. Second, we apply these criteria consistently across four domains: systems and synthetic biology, pharmacology and drug discovery, epidemiology, and neuroscience, together with worked case studies. Third, we frame current limitations, including multimodal integration, rigorous validation, and generalization to new biological systems, as an interlocking set of constraints and outline a future-oriented roadmap.

This is a narrative review with an explicit scope rather than a systematic survey. We searched PubMed, Web of Science, and Google Scholar for studies published predominantly between 2015 and 2026, using combinations of terms including “mechanistic model”, “mathematical model”, “machine learning”, “deep learning”, “physics-informed neural network”, “surrogate model”, “simulation-based inference”, and “digital twin”. Studies were selected for (i) direct integration of mechanistic knowledge with learning algorithms, (ii) methodological or biological influence, and (iii) coverage of the four target domains, yielding a corpus of approximately 95 to 100 references. This scope is a deliberate choice rather than an exhaustive survey. Areas closer to the authors’ clinical background, such as oral disease and oncology, are treated only as future application contexts (Section 5), because mature mechanism-AI integration studies in those fields remain scarce.

## 2. A Taxonomy of Integration: How Models and AI Collaborate?

**Figure 2:**
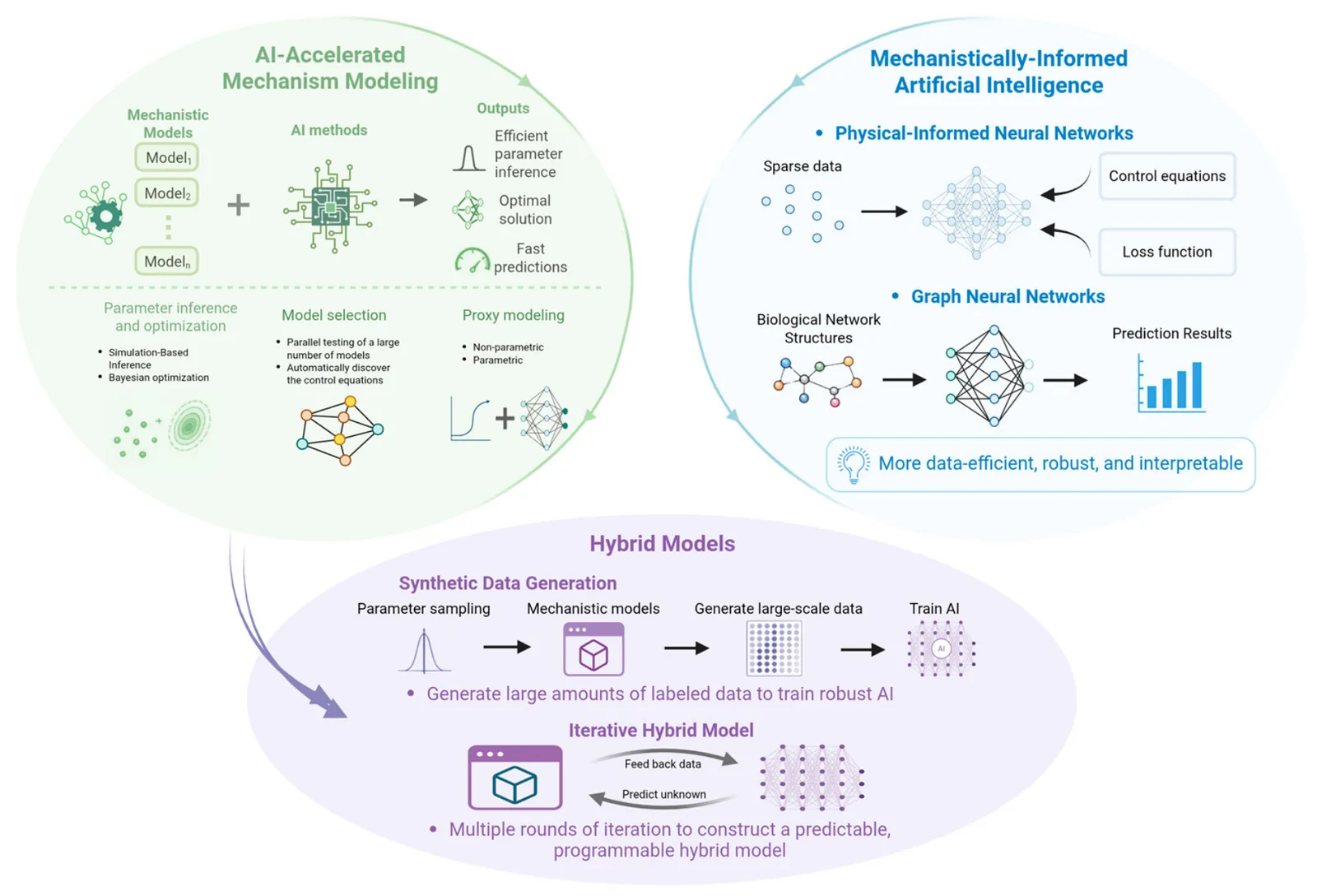
Major paradigms for the collaborative integration of mechanistic models and artificial intelligence. The figure depicts three distinct strategies arranged on a spectrum from mechanistic-driven (**left**) to AI-driven (**right**). **AI-accelerated mechanistic modeling** (**left**) uses AI as a computational accelerator for traditional mechanistic models: neural density estimators replace expensive Markov chain Monte Carlo sampling, surrogate models approximate slow biophysical simulations, and sparse regression discovers governing equations from data. **Mechanism-informed AI** (**center**) embeds known biological and physical laws into AI architectures: physics-informed neural networks encode governing equations as loss-function constraints, while graph neural networks rely on relational inductive biases that reflect known interaction topologies. **Iterative hybrid models** (**right**) establish closed-loop workflows in which generative AI proposes hypotheses, mechanistic models check proposals for consistency with assumed laws, and discrepancies feed back to refine both components. These paradigms are not mutually exclusive. They are points along a continuum, with increasing bidirectional influence between mechanistic knowledge and data-driven learning. (Created with Biorender.com).

Integrating mechanistic models with AI is not one task but a family of strategies. The approaches differ in how the two sides collaborate: AI accelerates traditional modeling, mechanistic knowledge guides AI, or the two merge into hybrid systems that blur the line between them (Figure 2). Each paradigm sits at a different point on a spectrum that runs from purely mechanistic to purely data-driven, and each fits a particular class of biological questions. Throughout this review, artificial intelligence (AI) denotes the broad field of computational systems that perform tasks normally requiring human intelligence; machine learning (ML) is the subset of AI that learns mappings from data rather than executing hand-written rules; and deep learning is the subset of ML based on multilayer neural networks. A mechanistic model means a model whose variables, parameters, and equations represent hypothesized biological entities and processes, whereas a purely data-driven model learns statistical mappings without such representational commitments. We assign each study to a paradigm using three criteria: which component carries the predictive burden, where mechanistic knowledge enters (as an accelerator target, as an architectural or loss-function constraint, or as a closed-loop partner), and the directionality of information flow. Studies instantiating more than one paradigm are classified by the dominant direction of flow.

### 2.1. AI-Accelerated Mechanistic Modeling

AI now acts as a high-throughput assistant, removing the main bottlenecks from traditional modeling workflows.

Estimating the parameters of ordinary differential equation (ODE) models is a classic inverse problem. Traditional Markov chain Monte Carlo (MCMC) methods generate samples that approximate the target posterior distribution through a random walk governed by a Markov chain. Their efficiency depends heavily on the proposal distribution, and each step needs an acceptance-probability computation that costs real time. New observational data force the entire sampling pipeline to be re-run, which makes real-time or instantaneous inference impractical. Simulation-based inference (SBI) offers a way out [18]. Instead of sequential chain-based sampling, SBI trains a neural network as a conditional density estimator on large volumes of simulated data. The network learns the implicit statistical mapping between data and parameters and yields posterior estimates directly. Once trained offline, it supports amortized inference: a forward pass on any new input returns the corresponding posterior estimate, and statistical inference becomes both faster and more flexible.

This amortized scheme fits complex models in neuroscience [27] and population genetics [28] within seconds, enabling analyses that were previously impractical, and independent benchmarks confirm the accuracy and robustness of neural density estimators across model classes [29]. When simulations themselves are expensive, Bayesian optimization steps in: it picks the next query point in parameter space by maximizing an acquisition function, reaches a global optimum in a minimal number of function evaluations [30], and offers a sample-efficient route to parameter optimization.

Model selection is a central challenge in complex biological systems, especially when several competing mechanistic hypotheses, each formalized as a distinct structure, have to be told apart. AI brings complementary tools. Scalable model-selection algorithms can screen many candidate structures on the same dataset and pick the optimal solution quickly [31]. Classical information criteria such as the Akaike Information Criterion (AIC) balance fit against complexity, but they require iterative screening and assume the candidate model set is known [31]. The governing equations themselves can also be discovered automatically. Sparse Identification of Nonlinear Dynamics (SINDy), for example, applies sparse regression to a large library of candidate functions and selects the minimal set of terms needed to represent the data, yielding concise and interpretable differential equations that reverse-engineer the system’s dynamics [32].

Many high-fidelity biophysical models, such as those built on finite-element analysis or entity-based simulation, are too slow for extensive exploration. Surrogate models offer a practical solution: fast, data-driven approximators trained on input–output pairs sampled from the slow simulator. Two families are commonly distinguished.

Non-parametric surrogates, such as Gaussian processes (GPs), train efficiently on small samples and quantify predictive uncertainty [33]. GP surrogates have optimized cardiac electromechanical simulations under sparse experimental data and accelerated uncertainty quantification in spatial cell-population dynamics with only a few high-fidelity runs.

Parametric surrogates, such as neural networks (NNs), scale to high-dimensional, data-rich problems and capture complex nonlinear behavior. Deep network surrogates have emulated three-dimensional cardiovascular computational fluid dynamics (CFD) and hemodynamics, several orders of magnitude faster than traditional numerical solvers, which enables real-time clinical parameter estimation.

The two families are complementary and are increasingly combined into hybrid architectures for both expressiveness and reliability [26]. Recent work has coupled autoencoders or deep networks, used for spatial dimensionality reduction, with Gaussian processes to map high-dimensional tissue biomechanics while keeping rigorous confidence bounds.

### 2.2. Mechanism-Informed Artificial Intelligence

Mechanism-informed AI works the other way around. Established biological and physical laws guide and constrain the AI models, and the models become more data-efficient, more robust, and more interpretable. This is part of the larger program of embedding physical principles into machine learning, which has already delivered substantial gains in the physical sciences [34].

Mechanistic knowledge can be injected at three points of differing assumption strength. The strongest form writes governing equations directly into the objective or architecture, as in physics-informed neural networks. A weaker form embeds architectural priors such as interaction graphs, which restrict which variables can communicate without imposing quantitative laws. The weakest form injects knowledge through initialization, feature design, or knowledge embeddings. These forms trade the strength of the assumed mechanism against flexibility, and the case studies in Section 3.5 illustrate the difference.

Complex systems are expensive to analyze, and in the low-data regimes typical of biology, purely data-driven black-box models overfit and generalize badly. Physics-informed neural networks (PINNs) meet this problem directly: they fit limited data under the constraint of known physical laws [35] by writing the governing equations into the dual-objective loss function of the network [36]. This regularization, central to the broader physics-informed machine learning movement [26], forces solutions that agree both with the sparse data points and with the underlying physics or biology. The approach works well for forward and inverse problems in cardiovascular modeling [37] and tumor growth [38], where data are sparse but the governing laws are well established.

Fixed, general-purpose neural networks hit fundamental limits on complex biological systems [38]. One remedy is to build known biological structure into the architecture itself, and graph neural networks (GNNs) are built for exactly this. Their relational inductive biases let them operate directly on nodes and edges [39]. For a signaling pathway, for example, the GNN is constructed so that nodes are proteins and edges are the known interactions [40], and the network learns the functional consequences of these interactions from data. The number of parameters to learn shrinks sharply, and the resulting model is easier to interpret. The practical record backs this up. GNNs predict untested gene combinations in signaling networks more accurately than unstructured baselines [41], and in cell–cell communication inference they reach single-cell precision and expose microenvironmental differences that conventional methods smooth over [42].

### 2.3. Hybrid Models: Sequential Coupling and Iterative Closed Loops

#### 2.3.1. Sequential Hybrid Models: Mechanistic Data Generation for AI Training

Deep learning has driven major progress across many fields, but its optimization depends on massive labeled datasets, a resource that is notoriously difficult, or even impossible, to obtain directly from biological experiments [43]. This dependence is not absolute. Transfer learning, self-supervised pretraining, few-shot adaptation, and biology-specific foundation models already allow models pretrained on large unlabeled corpora to perform downstream tasks with comparatively few labels, and synthetic data provide a further route. Sequential hybrid models get around this scarcity by using established mechanistic models as synthetic data generators. Sampling parameters from a prior distribution and running forward simulations within the mechanistic framework produces millions of physically constrained, labeled examples on which robust AI classifiers or predictors can be trained.

Protein dynamics simulations, for instance, can train generative neural networks to propose complementary, biophysically plausible conformational states [44]. The sequential strategy works best when the fundamental physical or biochemical laws are well characterized, so that the mechanistic model establishes a reliable baseline before the AI scales up pattern recognition.

#### 2.3.2. Iterative Hybrid Models: Closed-Loop Optimization and Causal Validation

Sequential models transfer knowledge in one direction. Iterative hybrid models go further and let the AI and the mechanistic model talk to each other, creating a “virtuous cycle of discovery.” In systems biology, machine learning maps complex statistical relationships and makes rapid quantitative predictions from high-dimensional data, while mechanistic modeling keeps carrying the explicit biological knowledge and the causal explanations behind those relationships [25].

The scientific community remains cautious about “hallucinations” and about the limited interpretability of generative AI in scientific discovery, so the iterative framework adds a safeguard: the mechanistic model acts as a mechanistic consistency checkpoint that screens proposals against explicitly assumed laws [26]. This screening enforces consistency with the assumed mechanism; it is not proof that the assumed structure is causally correct. In the proposed workflow, a generative AI model is first trained on a multimodal perturbation dataset to predict the effects of untested experimental conditions. The predictions are not accepted at face value. A mechanistic model then examines the predicted causal mechanisms for consistency with the assumed laws, and the discrepancies, errors, and newly validated insights are fed back into both architectures. Several rounds of closed-loop iteration reduce error reciprocally, keep the AI’s statistical predictions grounded in biological reality, and eventually yield a hybrid model that is predictable, programmable, and verifiable. (Table 1).

## 3. Frontiers of Application: Where Synergy Creates Breakthroughs

**Figure 3:**
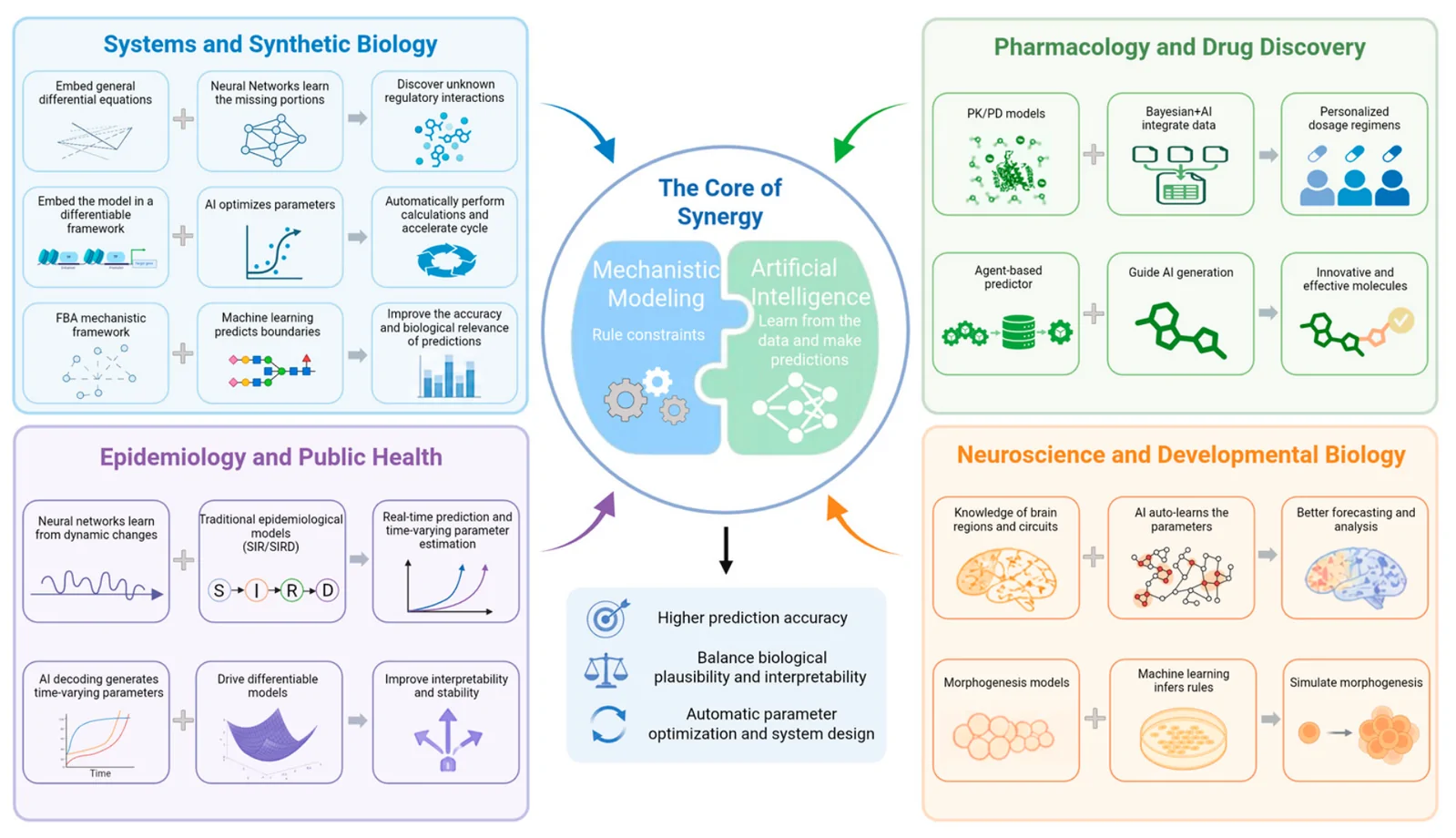
Representative applications of mechanism-AI integration in four domains: systems and synthetic biology, pharmacology and drug discovery, epidemiology, and neuroscience and developmental biology. Selected studies from each domain, including the in-depth case analyses of Section 3.5, are discussed in Section 3.1, Section 3.2, Section 3.3, Section 3.4 and Section 3.5.

The theoretical appeal of this synthesis is being confirmed by a growing list of practical successes across the life sciences (Figure 3), from genetic circuits to whole-brain dynamics.

### 3.1. Systems and Synthetic Biology

Gene regulation fits a “known-plus-unknown” knowledge structure, and hybrid models match that structure naturally. Generalized differential equations, in which neural networks stand in for the unknown terms, have been used to discover the missing regulatory interactions in cellular circuits directly from time-series data. The resulting universal differential equations are trained with local derivatives and adjoint methods against the observed time series, and symbolic regression then recovers symbolic equations that complete the regulatory structure [46]. Synthetic biology faces the opposite task: forward engineering. The Zygote system, written in Julia, provides differentiable programming with automatic differentiation over virtually all language constructs, without user intervention or restructuring of the computational phase [47]. Designers can build models of synthetic genetic circuits and of the cellular “host” chassis, then tune parameters collaboratively in the simulator by gradient-based optimization. The design–build–test–learn cycle accelerates, and the resulting designs perform more robustly when constructed.

Biotechnology rests on understanding and modifying cellular metabolism. Flux balance analysis (FBA) is a powerful mechanistic framework, but its single optimization objective limits predictive performance [48]. Machine learning models trained on multi-omics data, by contrast, predict metabolic fluxes with high accuracy yet cannot guarantee stoichiometric consistency. Synergistic approaches reconcile the two: reaction boundaries predicted by machine learning constrain the solution space of FBA and improve both the accuracy and the biological relevance of predictions [49]. Another design has neural networks learn the rules that govern metabolic pathways while the underlying model enforces biochemical laws such as mass balance. The dAMN model, built this way, achieves high predictive accuracy and generalization for bioprocess optimization [45].

### 3.2. Pharmacology and Drug Discovery

Graph neural networks that represent molecular and protein interaction networks are increasingly used to predict drug–target interactions (DTIs) accurately [50]. The GNN-based MHGNN, for example, exceeded 97% AUC and AUPR on the Luo benchmark, although a random split alone does not isolate the contribution of its structural priors (Section 3.5) [51]. Once a drug enters development, pharmacokinetic/pharmacodynamic (PK/PD) models describe its journey through the body and its effects on the target. The classical formulation uses mechanistic ordinary differential equations, and it has limitations [52]. Machine learning can help. By fusing prior and individual data with Bayesian methods and letting prediction errors adjust the weights automatically, hybrid models are rapidly personalized from sparse patient data [53], and individual dosage regimens are optimized.

Generative AI models propose novel molecular structures, but the structures may lack chemical meaningfulness or favorable pharmacological properties. A new frontier is to constrain the generation process with biophysical rules. Combining generative models with property predictors trained on physical simulations steers AI toward molecules that are both novel and therapeutically optimized [54]. MT-DiffGen is a typical example: a differentiable affinity predictor is supervised on experimental drug–target binding data, and a conditional diffusion generative model is guided by gradient feedback from that predictor, producing chemically valid molecules with high synthetic feasibility [55]. A related distinction separates affinity-guided generation from chemistry-native conditioning. VeGA-RX applies SMARTS-based functional conditioning and VeGA-SCX adds scaffold conditioning; both steer generation through chemical rules rather than a quantitative dynamical mechanism, so we class them as constrained or controllable molecular generation rather than mechanistic modeling in the strict sense [56].

Machine-learning models also integrate sequence, structure, and expression data with clinical variables to identify microRNA and other non-coding RNA biomarkers [57,58], to infer disease-associated regulatory relationships, and to support the design of small nucleic-acid therapeutics [59]. These applications extend the mechanism-informed paradigm to post-transcriptional and epigenetic regulation, although most current models remain predictive rather than coupled to explicit kinetic mechanisms.

### 3.3. Epidemiology and Public Health

Traditional epidemiological models have proved their value in disease transmission, but the COVID-19 pandemic exposed their limits, for instance, those of the classic Susceptible–Infected–Recovered (SIR) model [60]. These models give mechanistic insight but adapt poorly to real-time changes in human behavior [61]. Hybrid approaches use machine learning to keep SIR parameters up to date from real-time data streams. Coupling long short-term memory (LSTM) networks with the Susceptible–Infected–Recovered–Dead (SIRD) model and mobility data, for example, yields high-precision real-time predictions and time-dependent parameter estimates [62]. Physics-informed neural networks have also learned the temporal and spatial dynamics of disease transmission directly from case data [63].

Purely data-driven AI models are strong at short-term outbreak forecasting, since they learn complex spatiotemporal patterns, but they cannot predict new data under changing conditions [64]. Mechanistic models complement them by adjusting parameters dynamically. The most promising systems are not simple couplings. They use AI to decode observational data into time-varying parameters that drive the mechanistic model as it forecasts. EpiNode, a neural ODE + SIRS framework, addresses partial observability and non-stationarity and generalizes better [65]. End-to-end training is an alternative: variable functions learned by AI from the data are injected into a differentiable SEIR model and optimized together with it, which improves interpretability and robustness when conditions change [66].

### 3.4. Neuroscience and Developmental Biology

The brain may be the ultimate complex biological system. At the circuit level, hybrid models take a mechanistic framework and let AI learn the unknown parameters so that predictions track real-world data as closely as possible. Such models predict stimulus-evoked neural and behavioral responses better [67], and they train on experimental data extremely quickly and produce online predictions for new experiments [68]. At the whole-brain level, GNNs learn parameters from fMRI data under constraints provided by mechanistic knowledge of brain regions and functional structure, improving fMRI analysis [69].

How do cells self-organize into tissues and organs? Agent-based models (ABMs) and cellular automata offer powerful bottom-up mechanistic frameworks for simulating the process, yet the “rules” of agent behavior are usually unknown. Machine learning can infer them from time-lapse microscopy of living cells [70]. Conversely, AI-driven genetic algorithms can take features extracted from desired morphological patterns and optimize the outputs of neural networks and ABMs, yielding a hybrid framework that simulates morphogenesis [71]. Differentiable frameworks also recover interpretable gene networks through gradient optimization [72].

### 3.5. Representative Case Studies: What Does the Synergy Actually Deliver?

Three studies illustrate what the synergy contributes beyond a strong metric. Each is summarized by the same template: the biological problem, the mechanistic component, the AI component, the demonstrated comparison against a conventional approach, and the remaining limitation.

In drug discovery, the GNN-based MHGNN reached above −0.97 AUC and AUPR on the Luo drug-target-interaction benchmark. The result demonstrates predictive performance, but because the headline comparison does not isolate the structural priors, a fixed-architecture ablation that removes the mechanistic features, together with external validation, is still required to attribute the gain to mechanism. In pharmacokinetics, the hybrid model of Hughes and Keizer coupled a compartmental pharmacokinetic model to a machine-learning prior-correction layer and outperformed conventional maximum-a posteriori Bayesian estimation on the same data, though prospective clinical evaluation remains open. In epidemiology, the prospectively evaluated AI-augmented compartmental forecasts of COVID-19 in the USA and Japan used held-out future dates, illustrating both the value of prospective design and the distribution-shift problem under changing interventions.

High AUC values from random splits alone establish predictive performance but do not establish the value added by the mechanistic component. We suggest three minimum evidence standards for future claims of synergy: matched mechanism-free controls or ablations, temporally or externally held-out evaluation, and reported calibration and failure modes.

## 4. The Grand Challenges: Overcoming the Pitfalls

**Figure 4:**
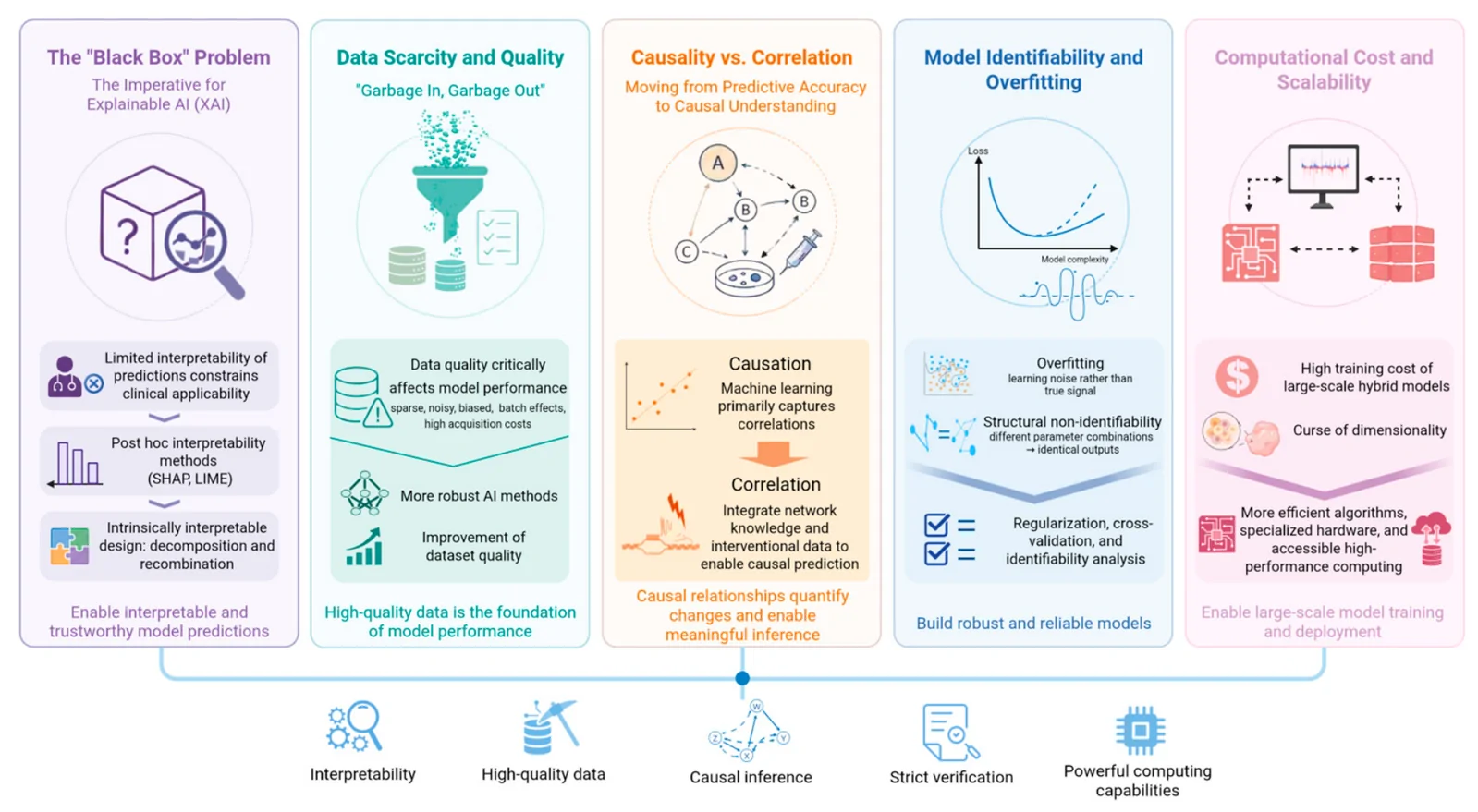
Key challenges and potential solutions in the integration of mechanistic models and artificial intelligence. The figure organizes seven interlocking obstacles along with corresponding mitigation strategies. (1) Interpretability: post hoc explanation tools (SHAP, LIME) provide attribution but require customization; explainable-by-design approaches (symbolic regression, concept bottlenecks) offer more principled transparency. (2) Data sparsity: transfer learning and synthetic data generation from mechanistic priors reduce dependence on large labeled datasets; community data standards and benchmark datasets improve model evaluation. (3) Causality: causal claims require intervention data and identifiability analysis; GEARS and CREOG generate candidate relationships that must be tested rather than causal conclusions. (4) Identifiability: strict regularization, cross-validation, and structural identifiability analysis prevent overfitting and ensure parameters reflect underlying biology. (5) Computational cost: algorithmic innovations (e.g., amortized inference, surrogate modeling), specialized hardware (GPUs, TPUs), and cloud computing resources address scalability bottlenecks. (6) Multimodal integration: modalities differ in noise, dimensionality, missingness, and platform bias, and aligning them across patients and biological scales requires nested submodels rather than naive aggregation. (7) Validation: leakage from preprocessing or overlapping patients, distribution shift, and miscalibration mean that reported performance often does not survive external or prospective testing. These challenges are interconnected (solving one often alleviates others) and must be addressed together. (Created with Biorender.com).

The path to full synthesis is littered with conceptual and technical obstacles, and naming them is a precondition for addressing them (Figure 4).

### 4.1. The “Black Box” Problem: The Imperative for Explainable AI (XAI)

As hybrid models grow larger neural-network components, they risk reintroducing the very problem they were meant to solve. If the reasons behind a prediction sit in millions of inexplicable weights, the prediction has limited clinical value [73]. The field of explainable AI (XAI) contributes tools such as SHAP [74] and LIME, which attribute a model’s output to its inputs. Post hoc explainability, however, usually needs to be customized for complex biological systems and is hard to quantify or evaluate [75]. A more promising direction is explainable-by-design modeling. Neural networks trained with symbolic regression, for example, decompose a complex problem into simpler ones, fit formulas with few variables to each, and recombine them into a single explainable symbolic expression [76]. The black-box problem is a property of particular model classes rather than of AI as a whole. Sparse linear models, generalized additive models, and tree- or rule-based methods are directly interpretable, and some deep models are interpretable by construction. Conversely, a complex mechanistic model with hundreds of unidentifiable parameters is not transparent in practice, and post hoc attribution of an association is not the same as recovering a biological mechanism.

A common design in current research splits the machine-learning pipeline into three iterative stages, training, evaluation, and optimization, and inserts additional datasets or prior knowledge at intermediate steps. SAGE-Prot for protein design is one such framework. On closer inspection, this task-oriented modular decomposition stays too coarse. A finer subdivision of the architecture into layers, such as input, concept, generation, and feedback, with further internal refinement, may be more effective. The concept layer, for instance, can be split into a concept bottleneck, a concept-value evaluator, and an input interface, which supports more robust concept ingestion, interpretation, and vectorization.

### 4.2. Data Sparsity and Quality: “Garbage In, Garbage Out”

Mechanism-based AI is more data-efficient than pure black boxes, but data-quality problems still hit it, since every model ultimately depends on the quality and quantity of its training data [77]. Biological data are often sparse, noisy, biased, burdened by batch effects, and expensive to acquire. That complicates analysis and integration and frequently leads to misinterpretation [78,79], which has stalled many ambitious AI projects. Solutions have to come from several directions: AI methods that tolerate small, imperfect datasets, such as transfer learning [80], and, at the community level, data standards, data sharing, and the generation of high-quality “ground-truth” benchmark datasets.

Dataset construction largely determines the quality of the trained model, and the mutational variability of biology is one of the major difficulties. Overly homogeneous data produce models that perform poorly in real-world settings because they cannot accommodate environmental fluctuation and heterogeneity, while too many extreme samples induce overfitting and compromise accuracy. Many high-quality datasets already exist, including imaging collections such as FOMO260K, MetaChest, IPATH, and SynthUterus ROI, which have greatly facilitated model development. Model robustness and dataset construction should advance in parallel.

For mechanistic and mechanism-informed models, the gold standard needs four properties. First, evaluation data must be independent in the biologically relevant sense, with patient-level, site-level, and ideally temporal splitting to prevent leakage from correlated samples, repeated visits, relatives, or preprocessing fitted on the full dataset. Second, benchmarks need matched mechanism-free controls and ablations so that gains are attributable to the mechanistic component rather than to capacity. Third, the ground truth must match the claim: held-out measurements for prediction, perturbation outcomes for intervention-level claims, and prospective patient outcomes for clinical use. Fourth, calibration is assessed alongside point accuracy. Community challenges with hidden test sets are the closest existing analog.

### 4.3. Causality vs. Correlation: From Predictive Accuracy to Understanding Causal Relationships

Standard machine learning learns correlations, not causal relationships [81,82]. It is useful to separate four levels of claim: predictive association, mechanistic interpretation, intervention-based inference, and formal causal discovery. Mechanistic models operate mainly at the second and third levels: they encode explicit causal assumptions, but agreement between their predictions and observations does not by itself validate the assumed causal structure, which must instead be probed through interventions and identifiability analysis. Perturbation datasets carry more causal information than observational associations, but accurate prediction of perturbation outcomes is still not equivalent to causal discovery. GEARS, for example, performs intervention-level prediction of unseen perturbation outcomes validated on held-out perturbations [41], which is not the same as recovering the true causal graph. Pearl’s framework supplied the mathematical language for this pursuit, and its integration with modern machine learning remains an active area of research.

A recent causal engine, CREOG, attacks causal questions in biology through an ontology-enriched knowledge graph. It generates causal triples augmented with confidence weights and textual provenance, which translates knowledge bases into a machine-readable format, and by association-rule mining it yields confidence-weighted, provenance-traceable candidate relationships rather than definitive causal results. CREOG also outlines three directions for causal problems: causal input representation, causal link algorithms, and causal inference output mechanisms. Treating the causal prediction engine as modules improves optimization efficiency and, to some extent, keeps the black box at bay.

### 4.4. Model Identifiability and Overfitting

Overfitting and identifiability are old challenges that hybrid models amplify. A complex mixed model with thousands of parameters can easily overfit sparse biological data, learning noise instead of signal and performing well on training data while failing to generalize. A subtler danger is structural unidentifiability: different parameter combinations produce the same model output, so one cannot tell which parameters reflect the underlying biology [83]. Strict regularization, cross-validation, and identifiability analysis are not optional extras. They are required for building robust models whose parameters can be meaningfully interpreted [84].

### 4.5. Computational Cost and Scalability

Training large-scale hybrid models is computationally demanding. A single PINN or SBI training run costs significant time and money, and a PINN with about 2.2 million parameters took 600 to 3500 min to train [85]. Moving from single cells to entire organs triggers the “curse of dimensionality,” with computational demands growing exponentially and straining resources [86]. This burden is well on its way to becoming a major bottleneck. Relief has to come from several fronts at once: more efficient algorithms, specialized hardware, and broad access to high-performance computing.

The seven challenges described above are not isolated problems but links in a chain. Data issues sit upstream. Sparse, noisy, biased data directly induce overfitting, confound causal identification, and raise computational costs, since complex architectures become necessary. Interpretability and causality together point to a lack of cognitive transparency: when parameters carry no clear meaning and the relationships are merely statistical, structural unidentifiability follows, and the model degenerates into curve fitting. Computational cost constrains the remedies at the resource level and forces researchers to trade model capability against verification sufficiency.

The cycle is self-reinforcing: data scarcity, model complexity, uninterpretability or non-causality, difficulty of verification, computational overload, and, finally, constraints on data enhancement. Future breakthroughs will require progress on algorithms, data infrastructure, and computing resources at the same time. Only by breaking this cycle can mechanistic models and artificial intelligence be integrated.

### 4.6. Integrating Heterogeneous Modalities and Biological Scales

Integrating heterogeneous data is a distinct challenge that operates at three levels. At the measurement level, modalities differ in noise, dimensionality, missingness, sampling rate, and batch or platform bias. At the representation level, integration requires cross-patient and cross-time alignment, joint latent-variable models, multimodal transformers, contrastive alignment, missing-modality training, and imputation. At the biological level, the molecular, cellular, tissue, organ, and organism span calls for nested mechanistic submodels rather than naive aggregation. Multimodal datasets also multiply leakage risks, because the same patient can appear in every modality, making patient-level splitting mandatory (Section 4.7).

### 4.7. Validation, Generalization, and Prospective Evaluation

Validation deserves the same attention as model design. Data leakage arises from preprocessing, feature selection, overlapping patients, and temporal information. Distribution shift appears across hospitals, assays, populations, and pathogen strains, so external cohorts and independent sites are the key transfer test. Calibration should be reported alongside discrimination, and epistemic and aleatoric uncertainty should be distinguished. The clinical standard is prospective evaluation of frozen models. The presumed generalization advantage of mechanism-informed models is an empirical hypothesis to be tested on shifted data, not a guarantee. We recommend reporting dataset provenance, the splitting procedure, component ablations, and failure modes.

## 5. The Next Horizon: Future Perspectives

**Figure 5:**
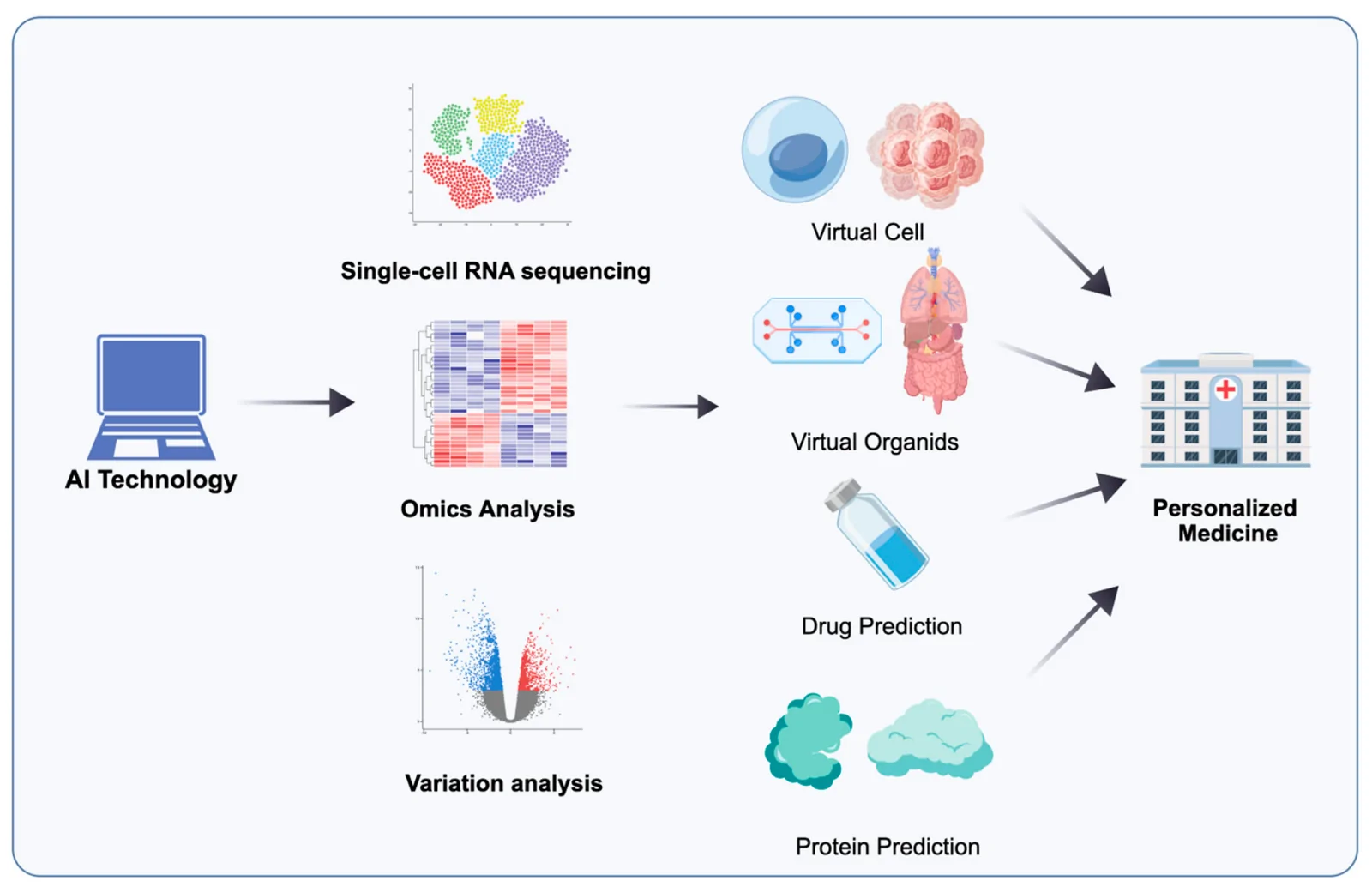
A roadmap from multimodal patient data to predictive and personalized medicine. Multi-omics, medical imaging, electronic health records, and wearable-sensor streams (**left**) are integrated through coupled mechanistic and AI model layers (**center**). The same workflow supports three longer-term endpoints (**right**): biological digital twins, causal discovery engines, and automated scientific discovery. The figure depicts the data-to-decision workflow; the three endpoints are aspirational and are discussed separately in Section 5.1, Section 5.2 and Section 5.3.

The challenges the integration faces double as a research agenda. Meeting them would realize three long-term visions: biological digital twins, causal discovery engines, and automated science (Figure 5).

### 5.1. Biological Digital Twins: Current Capabilities and Long-Term Goals

Physics, biology, and medicine are now interwoven, and interdisciplinary work can give biological digital twins strong quantitative support [87]. Physics reveals quantitative relationships between data, biology supplies mechanistic readings of clinical phenotypes and longitudinal records, and the two fields reinforce each other once mathematical models are in place.

An ambitious expression of this synergy in medicine is the “digital twin”: a personalized, multiscale computational model of an individual patient, continuously updated with their clinical data [88]. The model couples physiological mechanism knowledge with AI-driven personalization from genomics, imaging, and wearable-sensor data. It can simulate the patient’s future health trajectory, test treatment strategies computationally before they are applied, and provide a rational basis for personalized and preventive medicine, conditional on prospective validation [89].

Bio-digital twin systems let clinicians run operations in a virtual environment, such as component analysis, disease identification, and clinical simulation. That widens the safety margin in clinical medicine, and real-world care becomes more intelligent and precise. The demands on the mathematical models and the AI training of each module rise accordingly, and the larger challenge is integrating all the systems into an automated whole.

These virtual systems presume foundational models capable of simulating cellular behavior and tissue-level responses, and recent work has begun to supply the building blocks. Deep generative models reconstruct disease-progression trajectories from time-series single-cell data and enable in silico perturbation of cellular states so that therapeutic outcomes can be predicted in a purely virtual environment [22]. Integrative frameworks that link single-cell transcriptomic profiles with patient survival identify high-risk subpopulations and simulate patient-specific gene perturbations [24]. AI-enhanced organoid platforms, which combine multi-omics, morphology, and functional assessment, provide scalable in vitro models that can act as virtual organoid substrates for drug screening and disease simulation [23]. Together these advances move biological digital twins from concept toward reality, assembled from modular, computationally integrated components that operate across molecular, cellular, and tissue scales.

To state it plainly, this is what exists today and what remains aspirational. Current systems demonstrate key components: deep generative models reconstruct single-cell trajectories and permit in silico perturbation, integrated single-cell and bulk frameworks link cellular states to patient-level risk, AI-enhanced organoids provide measured in vitro substrates, and physics-based organ twins, for example in cardiology, already support device and therapy design. A continuously updated, multiscale, patient-specific twin validated for autonomous clinical decisions, by contrast, remains a future goal. In silico interventions should therefore currently be read as decision support and hypothesis generation, not as validated substitutes for clinical trials or clinical judgment.

### 5.2. Causal Discovery Engines

Interpretable associations, even with attribution methods, are not causal discovery. Mathematical and AI models can interrogate the interrelations among data points, filter out interference from high-dimensional factors, and quantify how low-dimensional factors track disease progression, but establishing cause requires intervention-based inference [90]. Most current digital models, however, stop at information reception and analysis: they work on pre-prepared data and results under supervised learning and cannot anticipate what happens next when independent variables change. Precise medical regulation becomes harder as a result. An effective causal discovery engine remains a long-term goal. It should combine real-time dynamic data analysis to construct regression models, prior knowledge to reduce false causal inferences, robust mathematical models and algorithms, prediction of clinical phenotypes from changes in independent variables, digital-twin systems for personalized forecasting, and built-in interpretability and confidence quantification. Reaching that goal will take sustained interdisciplinary effort.

We envision integrated “causal discovery engines” that combine large observational datasets, AI-driven hypothesis generators, and differentiable mechanistic models. Such an engine proposes a candidate causal relationship, then uses that model to design experiments with maximum information content and minimum cost to test it, and feeds the results back into the system, closing a loop that iteratively tests and refines a candidate causal map of biological networks [91,92].

### 5.3. Automated Scientific Discovery

Artificial intelligence can already “predict the future” in a narrow sense: it makes mechanical inferences from prior knowledge with high accuracy [93], and it can refine its reasoning with laboratory data, reducing noise and error [94]. This signals a gradual shift in what AI is. It is no longer merely a pattern-recognition tool but an iterative, self-correcting engine for scientific reasoning, one that extracts patterns from prior knowledge, recalibrates with experimental feedback, and approaches real-world dynamics in a virtual environment.

Looking further ahead, AI agents with greater scientific autonomy become conceivable. They could scan the literature for knowledge gaps, formalize hypotheses into hybrid models, propose and even execute robotic experiments to collect data, and interpret the results and publish new discoveries [95]. Full automation remains a distant goal, but pursuing it will produce powerful tools that enhance and accelerate human scientific creativity [96].

### 5.4. A Call for a New Generation of “Bilingual” Scientists

From simple mathematical models to today’s large models, scientists are pushing the limits of what can be computed in biology and, at the same time, the limits of what machine learning can cover [96,97]. The “Society 5.0” concept has put serious demands on AI in medicine [98]. Digital healthcare, to become real, needs full use of resources, deeper interdisciplinary integration, and institutional reform. It also needs a cultural and educational shift. The walls between experimental biology, mathematical theory, and computer science have to come down. We need a new generation of “bilingual” scientists, fluent in the language of molecules and in the language of models, and graduate programs, funders, and collaborative platforms must be restructured to support and reward interdisciplinary work.

This demands more than technical skill. It requires scientists fluent in molecular biology and mathematical formalisms, with the patience to sit with complexity without premature reduction. The silos separating computation and experiment need to come down, open infrastructures for data, models, and protocols need to be built, and negative results and mechanistic null hypotheses should count as much as predictive successes.

The destination, a science of living systems that is both predictive and comprehensible, will not be reached by AI alone or by mechanism alone. It will be built by researchers who combine prediction with mechanism, by communities that maintain open data, models, and benchmarks, and by institutions that reward such interdisciplinary work. We hope the taxonomy, evidence standards, and roadmap offered here provide a useful structure for that effort.

## 6. Conclusions

Mechanistic modeling and machine learning answer different questions, and the value of combining them lies in what each supplies to the other. Mechanistic knowledge constrains a hypothesis space that data alone cannot, and learning algorithms explore that space far faster than hand-tuning allows. The taxonomy proposed here separates the three ways this happens, and the case studies in Section 3.5 make the standard for claiming success explicit: a gain has to survive an ablation that removes the mechanistic component, and it has to hold on data that were not used to build the model. Where that standard has been met, the results are encouraging. Where it has not, high benchmark scores are difficult to interpret.

The obstacles described in Section 4 are coupled. Sparse and biased data drive overfitting and weaken causal claims, unidentifiable parameters undermine interpretability, and computational cost limits the validation that would resolve these problems. Progress on any one of them therefore depends on the others, which is why we treat them as a single agenda rather than a list. Biological digital twins, causal discovery engines, and automated discovery, the three directions sketched in Section 5, all presuppose that agenda being met. None of them is close to routine clinical use, and the honest position is that in silico results currently support decisions rather than replace them. Building models that are predictive, interpretable, and causally grounded will require researchers trained in both molecular biology and computation and institutions willing to reward that combination.

## Figures and Tables

**Table 1:** Comparison of three major paradigms for the collaborative integration of mechanistic models and artificial intelligence.

Paradigm	Core Strategy	Representative Methods	Key Advantages	Biological Applications	References
AI-accelerated mechanistic modeling	AI assists traditional mechanistic models by solving computational bottlenecks	Simulation-based inference (SBI), Bayesian optimization, SINDy, surrogate models (Gaussian processes, neural networks)	Dramatically reduces computational cost; enables rapid parameter inference and model selection; handles high-dimensional parameter spaces	Parameter estimation in neuroscience and population genetics; automatic discovery of governing equations from time-series data	[18,27,28,32,33]
Mechanism-informed AI	Biological and physical laws guide and constrain AI models	Physics-informed neural networks (PINNs), graph neural networks (GNNs) with structural priors	Improves data efficiency and generalizability; enhances interpretability; reduces overfitting in sparse-data regimes	Cardiovascular modeling, tumor growth simulation, signaling pathway prediction, cell–cell communication inference	[35,37,38,41,42]
Sequential and iterative hybrid models	Mechanistic models and AI engage in collaborative, closed-loop refinement	Synthetic data generation + AI training, differentiable programming, hybrid neural-ODE frameworks	Creates a virtuous cycle of discovery; enables continuous model improvement; integrates correlative and causal insights	Protein conformational sampling, metabolic flux prediction, programmable synthetic biology circuits	[45]

Note: ODE, ordinary differential equation; SBI, simulation-based inference; SINDy, sparse identification of nonlinear dynamics; PINN, physics-informed neural network; GNN, graph neural network.

## Data Availability

No new data were created or analyzed in this study. Data sharing is not applicable to this article.
